# The research foundation for COVID-19 vaccine development

**DOI:** 10.3389/frma.2023.1078971

**Published:** 2023-03-24

**Authors:** Komi S. Messan, Pawel P. Sulima, Dolan Ghosh, Jonathan Nye

**Affiliations:** ^1^Data Analytics and Research Branch, National Institutes of Health, National Institute of Allergy and Infectious Diseases, Office of Strategic Planning Initiative Development and Analysis, Rockville, MD, United States; ^2^National Institutes of Health, Office of the Director, NIH Office of Extramural Research, Bethesda, MD, United States

**Keywords:** COVID-19 vaccine, natural language processing, text mining, machine learning, citation analysis

## Abstract

The development of effective vaccines in <1 year to combat the spread of coronavirus disease 19 (COVID-19) is an example of particularly rapid progress in biomedicine. However, this was only made possible by decades of investment in scientific research. Many important research commentaries and reviews have been provided to describe the various contributions and scientific breakthroughs that led to the development of COVID-19 vaccines. In this work, we sought to complement those efforts by adding a systematic and quantitative study of the research foundations that led to these vaccines. Here, we analyzed citations from COVID-19 vaccine research articles to determine which scientific areas of study contributed the most to this research. Our findings revealed that coronavirus research was cited most often, and by a large margin. However, significant contributions were also seen from a diverse set of fields such as cancer, diabetes, and HIV/AIDS. In addition, we examined the publication history of the most prolific authors of COVID-19 vaccine research to determine their research expertise prior to the pandemic. Interestingly, although COVID-19 vaccine research relied most heavily on previous coronavirus work, we find that the most prolific authors on these publications most often had expertise in other areas including influenza, cancer, and HIV/AIDS. Finally, we used machine learning to identify and group together publications based on their major topic areas. This allowed us to elucidate the differences in citations between research areas. These findings highlight and quantify the relevance of prior research from a variety of scientific fields to the rapid development of a COVID-19 vaccine. This study also illustrates the importance of funding and sustaining a diverse research enterprise to facilitate a rapid response to future pandemics.

## 1. Introduction

The rapid production of vaccines that effectively combat severe acute respiratory syndrome coronavirus 2 (SARS-CoV-2) was unprecedented in the history of infectious disease treatment development (Bok et al., 2021; Conti, 2021; Kuter et al., 2021). Efficacious vaccines were developed in <1 year from the identification of the virus (Fauci, 2021). Prior to the spread of coronavirus disease 19 (COVID-19), the disease caused by the SARS-CoV-2 virus, the average vaccine required over 10 years to develop (Pronker et al., 2013). Even the accelerated response to the West African Ebola outbreak in 2014 required 5 years from the start of Phase 1 trials to produce a vaccine (Le et al., 2020). Facilitating the rapid response to COVID-19 has been the groundwork laid in earlier basic, preclinical, and clinical research—particularly in the fight against HIV—described in scientific journal editorials and the popular press. These articles cite the contribution of individual scientists such as Drew Weissman, Katalin Karikó, Barney Graham, Peter Kwong, Jason McLellan, Kizzmekia Corbett, Andrew Ward, Dan Barouch, and several of their close associates (Allen, 2020; Johnson and Bernstein, 2020; Fauci, 2021; Johnson, 2021a,b,c; Kolata and Mueller, 2022).

These anecdotal accounts serve as useful and important illustrations of the importance of sustaining a diverse research enterprise, encompassing all phases of research from basic studies through clinical trials. While the scientists mentioned in these articles might be among the COVID-19 “vaccine vanguard,” the work of thousands of scientists contributed to the coronavirus vaccines (Johnson, 2021c). In addition to research to develop an HIV vaccine, COVID-19 vaccine development built on earlier research to prevent diseases such as respiratory syncytial virus (RSV), Middle East respiratory syndrome (MERS) and severe acute respiratory syndrome (SARS) (Fauci, 2021).

These published accounts do not present a broad, systematic, and quantitative assessment of the contributions of earlier research to COVID-19 vaccine development. In this paper, we use several methods to more objectively quantify the relative contributions of different areas of study to COVID-19 vaccine research. We use a curated database of research articles categorized into areas of research using natural language processing (NLP) to identify the areas of expertise of some of the most prolific authors of COVID-19 vaccine research, reaching beyond the individuals highlighted in anecdotal accounts. Using this same method, we examine the categories of research cited by COVID-19 publications. Finally, we use topic modeling to examine in more detail the type of prior research cited in specific areas of COVID-19 vaccine research.

## 2. Methods

### 2.1. COVID-19 publication data collection

To serve the analytic needs of the COVID-19 research community, the National Institutes of Health (NIH) Office of Portfolio Analysis developed a publicly available, comprehensive, and expert-curated portfolio of COVID-19 publications and preprints based on the COVID-19 Open Research Dataset (Lu Wang et al., 2020; Santangelo, 2020). COVID-19 publication data were downloaded from the NIH website using the iSearch platform at https://icite.od.nih.gov/covid19/search/. To ensure the quality of the analysis, we excluded all preprints as they have not undergone peer review. Furthermore, a search query was developed that used PubMed Medical Subject Headings (MeSH) to identify and filter for COVID-19 publications related to vaccines. Review articles were excluded from the dataset as they don't represent original research and many of the most relevant references contained therein would themselves have been included in the search results. Finally, non-research article types were excluded from the dataset using PubMed publication type tags.

The final dataset extracted from iSearch included 6,946 COVID-19 vaccine-related documents. For each of these publications, its title, abstract, authors, cited references (and their PubMed identifiers, PMIDs), and MeSH conditions were extracted for analysis. The set of MeSH conditions is a subset of words and phrases in the NLM MeSH tree and they are derived using natural language processing, as described in the iSearch User Guide (National Institutes of Health, 2021a):

*Conditions are diseases, disorders, syndromes, illnesses, or injuries that are automatically extracted from titles and abstracts using natural language processing software that identifies phrases and synonyms along with their associated Medical Subject Headings (MeSH) semantic type*.

### 2.2. Subsequent data collection and analysis

Three primary analyses were based on this set of COVID-19 publications: ten-year publication histories of the most prolific authors of the COVID-19 publications were gathered and analyzed to determine areas of expertise; the literature cited by the COVID-19 articles was categorized by its disease relevance; and we examined the disease relevance of the cited literature within subfields of COVID-19 research identified by topic modeling the COVID-19 articles.

#### 2.2.1. Author expertise in COVID-19 vaccine research

One way to understand the foundation of COVID-19 vaccine research is to characterize the expertise of researchers who have published in this area. We used authors' publication histories in the 10 years prior to the COVID-19 pandemic to identify their area of expertise. To identify each author's publications, we used the Web of Science “Author Search” disambiguation algorithm (Dona, 2022).

While the Author Search functionality made gathering author histories easier, manual curation to create complete and accurate bibliographies was still required. Performing this for all authors of the 6,946 COVID-19 papers was prohibitively labor-intensive. The task was made more manageable by limiting the analysis to a subset of only the most prolific authors. We identified all (co)authors of the 6,946 COVID-19 vaccine-related articles extracted from iSearch. From this set, we identified the most prolific authors by selecting only those that had at least ten COVID-19 vaccine papers, resulting in a subset of 141 authors. Together, these researchers authored 730 papers (10.5%) of the COVID-19 publications.

An initial list of each author's articles published in 2010 through 2019 was collected from the Web of Science and Scopus databases. These initial publication histories were fact-checked by examining consistency in affiliated institutions and broad areas of research. Further confirmation of the accuracy of authors' publication history was performed by inspecting secondary sources of information such as author-curated publication lists on institution websites, publication search by ORCID ID, and LinkedIn profiles.

Medical conditions associated with each author's publications were obtained from an internal NIH version of iSearch accessible to NIH staff (National Institutes of Health, 2021b). The conditions in iSearch are defined in the iSearch User Guide (see COVID-19 publication data collection section above), and they consist of MeSH terms from various levels of the MeSH hierarchy. Related conditions were then grouped into broader categories of research. For example, viruses in the same genus like dengue, West Nile, and Zika were grouped together into the Flavivirus category and different cancer types are all grouped within the broader cancer category. An author was considered to have expertise in a specific research area if they had at least five publications with that specific MeSH condition. The accuracy of these expertise assignments was further confirmed using information about each author from publicly available websites.

#### 2.2.2. Disease relevance of literature cited by COVID-19 vaccine research

To identify the research area of the literature cited by COVID-19 vaccine research we first extracted all the cited publications from our initial set of 6,946 research articles. We then obtained the conditions associated with each of the resulting 68,352 cited publications from the iSearch database. Each condition was then mapped to a disease category as in author expertise in COVID-19 vaccine research section.

#### 2.2.3. Topic modeling to identify subfields within the COVID-19 vaccine literature

The open source software Jupyter-Python 3.9 Notebook (van Rossum and Drake, 2000; Kluyver et al., 2021) and R Version 4.0.4 (Team, 2020) were used to identify research subfields within the COVID-19 vaccine literature.

*Text Preprocessing*. The title and abstract of each of the 6,946 COVID-19 vaccine-related publications were concatenated, and the resulting text preprocessed by removing punctuation and stop words. The text was then lemmatized using the Python package spaCy (Honnibal, 2017).

*Clustering*. We performed topic modeling on the resulting corpus using BERTopic (Grootendorst and Reimers, 2020). BERTopic is a technique that uses Latent Dirichlet Allocation and leverages transformers [see transformers description in Wolf et al. (2019)] and class-based term frequency-inverse document frequency (c-TF-IDF) to create dense clusters allowing for easily interpretable topics while keeping important words or group of words in the topic descriptions (Grootendorst, 2020). BERTopic attempts to assign each document in the corpus to a single topic cluster. If a document is unable to be assigned to any cluster, it is classified as an outlier by BERTopic and thus not incorporated into any topic cluster.

We note that the size of the outlier cluster depends on both the quality and size of the dataset as well as the BERTopic parameters used. For our analyses, we set the n-gram range parameter to [2, 3] (i.e., two- and three-word combinations were used for the text vectorization). The minimum topic size parameter, which indicates the minimum documents per topic cluster, was initially set to 50.

An iterative application of BERTopic was used to minimize the number of outlier documents. After the first pass of BERTopic, outlier documents were submitted to a second analysis with the minimum topic size reduced to 40. A third BERTopic modeling was conducted on the outliers from the second pass using a minimum topic size of 20. All other BERTopic parameters were kept at their default value. All results from the machine-driven analysis were manually validated by a subject matter expert. We note that prior to using BERT, we have attempted K-means clustering technique that failed at clustering the documents into interpretable topics. Also, in previous studies (Benitez-Andrades et al., 2022; Bilal and Almazroi, 2022) the authors observed that BERT-based classifiers outperform bag-of-words approaches. Though BERT-based models can be computationally expensive (Bhattacharjee et al., 2020), we utilized BERTopic to have a better accuracy in classifying documents into interpretable topics.

#### 2.2.4. The disease relevance of research cited within COVID-19 subfields

We examined the frequency with which medical conditions, identified using the method in disease relevance of literature cited by COVID-19 vaccine research section, were cited within each of the COVID-19 subfields identified using the topic modeling described in topic modeling to identify subfields within the COVID-19 vaccine literature section. To control for differences in the number of articles falling in each of the topic categories and to identify any conditions that were cited disproportionately within a topic, we calculated a relative citation frequency by comparing the observed frequency distribution of conditions cited within each topic to their expected frequencies based on the total number of times a condition was cited across all topic areas and the number of publications in each topic:


$$RCF_{ij}=\frac{c_{ij}}{c_{j}\times \frac{p_{i}}{p}}$$

where:

*p* = 6,946, the total number of publications across all topics,

p_*i*_ is the total number of publications in topic category *i*,

c_*j*_ is the total number of cited articles related to condition *j*, across all topic areas,

c_*ij*_ is the observed number of citations to condition *j* within topic *i*,

(c_*j*_ x p_*i*_/p) is the expected number of citations to condition *j* within topic *i*, and

RCF is the relative frequency of citations to condition *j* within topic *i*.

From these RCFs, a heatmap was generated using the R package heatmaply (Galili et al., 2018) with the scale transformation applied to rows of the heatmap.

### 2.3. Methodological limitations

The particular datasets and analysis methods chosen for a study can place limitations on the validity and generalizability of the results. For this study, these include the following considerations.

First, since the beginning of the pandemic, more than 30 vaccines have been approved for use worldwide (Klobucista, 2022). Including a variety of different types such as viral vector vaccines, protein-based vaccines, and mRNA vaccines (Li et al., 2021). These different efforts have drawn on their own research corpora that will be represented to varying degrees in the database used in this study.

A second consideration is the collection of peer-reviewed articles in the iSearch COVID-19 portfolio is based, in part, on PubMed, whose primary resource is the MEDLINE bibliographic database. While MEDLINE is an extensive collection of published research in the biomedical sciences, it cannot capture all published articles related to COVID-19. The reliance on MEDLINE in the current study could introduce a potential bias, but it's not clear what the nature of this bias may be and whether it affected our results. However, in an analysis of 50 systematic reviews of therapeutic interventions, only one instance was found in which supplementing PubMed with additional data sources would have changed a review's outcome (Halladay et al., 2015). In addition, with respect to NIH-supported research, coverage in MEDLINE should approach 100 percent as a result of NIH's open access policy which, since 2008, has required deposition of all NIH-funded manuscripts in PubMed Central.

A final consideration is the use of iSearch to identify medical conditions and vaccine-related research. At present, there is little documentation of the categorization methods used by iSearch to identify diseases beyond a brief description in the iSearch User Guide, quoted in COVID-19 publication data collection section above.

As a result, there may be limits on the generalizability of our findings. It is likely that using other data sources also would be limited but in ways that are difficult to predict. A comparison among different data collection approaches would help to identify more precisely the limits of generalizability.

## 3. Results

### 3.1. Author expertise in COVID-19 vaccine research

**Figure 1:**
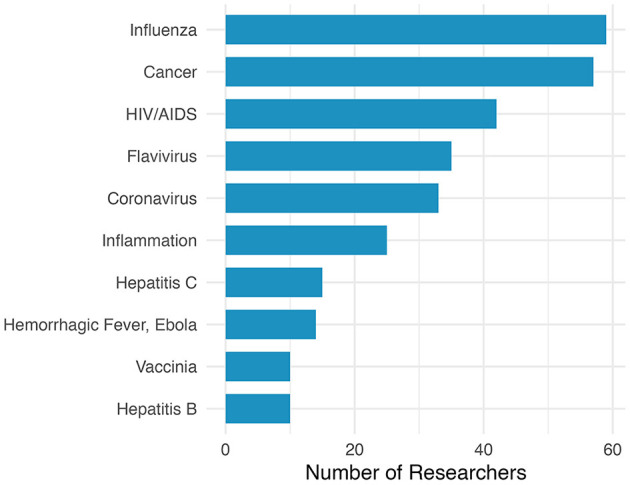
Research expertise of COVID-19 vaccine top authors.

The size and scale of the COVID-19 pandemic led to a rapid response from the scientific community. Indeed, vaccine research and development started almost immediately upon the identification of the virus. Although coronavirus researchers were heavily involved, the unprecedented nature of the pandemic drew investigators from a wide variety of different fields to aid in COVID-19 vaccine research. In order to better understand the areas of expertise of these researchers we analyzed their publication histories. Briefly, MeSH conditions associated with an author's publications from the previous ten-year period were used to determine their area of expertise. An author was determined to have expertise in a research area if they had at least five publications in a given field. Figure 1 is showing the 10 most prevalent areas of expertise of the most prolific COVID-19 vaccine authors. Our findings show that the most prolific authors had experience in fields such as influenza, cancer, and HIV/AIDS. Interestingly, coronavirus was only the fifth most frequent research field. As expected, eight of the top ten areas were infectious diseases (see Figure 1). However, both cancer and inflammation research were also heavily studied research areas.

### 3.2. Disease relevance of literature cited by COVID-19 vaccine research

**Figure 2:**
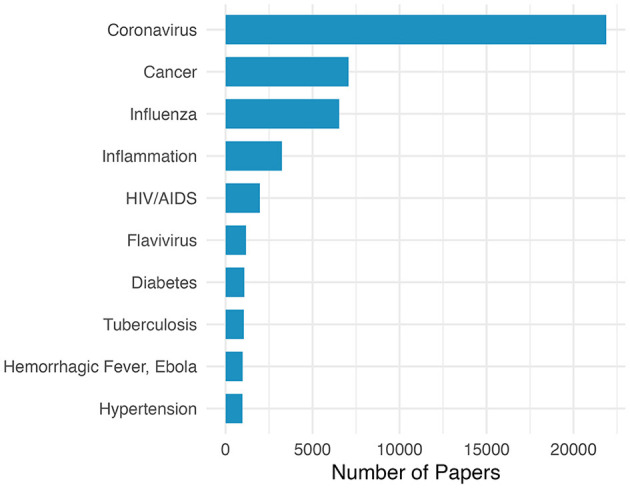
MeSH conditions most frequently associated with research articles cited by COVID-19 vaccine publications.

To gain insight into the scientific areas that contributed the most to COVID-19 vaccine research and development, we performed a citation analysis. In total, the 6,946 COVID-19 vaccine-related articles we identified cited 68,352 unique publications. The most frequently cited research areas were identified using the MeSH conditions extracted from this set of cited articles. Figure 2 shows the ten most cited research areas. Interestingly, although the top authors' expertise was more likely to be in areas outside of coronavirus research, as shown in Figure 1, our citation analysis revealed that coronavirus publications were cited 3.1 times more than any other research field. Many of the most cited research areas overlap with those found in our top authors' expertise including cancer, influenza, and HIV/AIDS. In addition, the majority of citations represent infectious disease fields. Moreover, our analysis also revealed a significant number of publications cited from non-infectious disease fields like cancer, inflammation, diabetes, and hypertension.

### 3.3. Clustering to identify major topic areas in COVID-19 vaccine research

To understand in more depth how the cited literature in Figure 2 contributed to COVID-19 vaccine research, we examined linkages between the disease relevance of the cited literature and subcategories of this larger research area. To further characterize the topics within the COVID-19 vaccine literature, we used a topic clustering algorithm, BERTopic, to identify groups of related documents based on the title and abstract of the publications. Using this method, 19 different topic clusters were identified (see Table 1). The 11 largest topics account for 90% of all publications including studies of COVID-19 infection, vaccine design, and novel therapeutics, as well as policies, practices, and regulatory and psychosocial issues surrounding the pandemic and vaccination. In addition, some studies focused on specific subpopulations, such as COVID-19 infection and vaccination in children or immunosuppressed patients.

**Table 1:** Identification of major topic areas in COVID-19 vaccine-related publications.

**Cluster**	**Title**	**Total**
**1**	**Assessment of novel vaccines and therapeutics**	**1,214**
2	Public health policies and practices for COVID	960
3	Analysis of COVID-19 infection, vaccination, and plasma therapy	773
4	Psychological and social determinants of COVID vaccine uptake	709
5	Structure and function of the spike protein and neutralizing antibody targets	484
6	Regulatory, ethical, and logistical considerations of COVID vaccination	474
7	Designing novel therapeutics through *In Silico* modeling	375
8	Risk factors for severe infection and potential therapies	354
9	Epidemiology and public health mitigation measures	330
10	COVID vaccine and therapeutic clinical trials	290
11	Immunization policy and practices	272
12	Epitope based vaccine design	193
13	COVID vaccination public health policies	123
14	BCG vaccination for COVID	96
15	COVID vaccines in pre and postnatal periods	93
16	Thrombotic events associated with COVID vaccines	69
17	COVID vaccination in immunosuppressed patients	61
18	Risks for pediatric COVID infection and vaccination	41
19	Data analytics, machine learning and mathematical modeling	35

Cluster indicates the topic cluster number, Title is the title of the topic cluster, and Total is the total number of documents under each cluster.

### 3.4. The literature cited within COVID-19 topic areas

**Figure 3:**
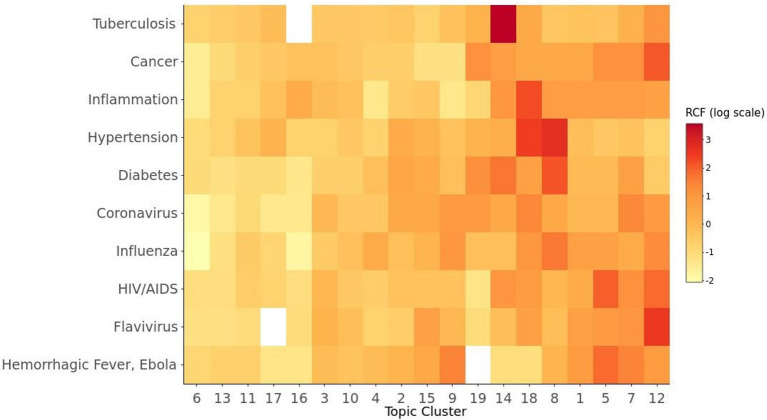
Mapping of COVID-19 research topics to conditions studied in the cited literature. RCF denotes the Relative Frequency of Citations. The titles for cluster 1 through 19 are as elaborated in Table 1.

A more detailed understanding of the foundations of COVID-19 vaccine research might be found by identifying relationships between these specific topic clusters and the literature each one cites. This type of analysis can show quantitatively how prior research in a specific field was used to inform particular topic areas within COVID-19 vaccine research. Therefore, we examined the frequency with which the MeSH conditions shown in Figure 2 were cited within each of the topic areas in Table 1. Figure 3 is a heat map showing the relative citation frequency (RCF) on a log scale of the MeSH conditions within the 19 topic areas. Darker colors are associated with higher relative frequencies. For example, topic 12, Epitope-Based Vaccine Design, drew heavily on HIV/AIDS literature as well as flavivirus and cancer focused publications. Topics 5 and 7—research on the COVID-19 spike protein, neutralizing antibody targets, and *in silico* modeling for drug discovery—drew heavily on research in some of the same disease areas, but also Ebola virus research. Moreover, research on inflammation, hypertension, and diabetes, contributed heavily to Topics 8 and 18—the study of various risk factors for COVID-19 infection and vaccination. Finally, prior research related to tuberculosis was cited very frequently by articles within the topic cluster 14, BCG [Bacille Calmette-Guérin] Vaccination for COVID-19. Many of the articles in topic 14 addressed the relationship between BCG, a vaccine currently used to prevent tuberculosis, and COVID-19 outcomes—in particular, the potential for BCG vaccination to mitigate the effects of COVID-19.

## 4. Discussion

It's important that we analyze and reflect on the research community's response to the pandemic so that we can gain insight into how to best prepare for any future events. Timely analysis of this data is essential since the foundational research and training of investigators needed to respond to novel outbreaks can take many years or even decades to develop. In this study, we were able to use multiple methods to quantify the relative contributions of different areas of researcher expertise, the focus of prior foundational research, and the relationships of these to specific areas of COVID-19 vaccine research.

Our analysis of the most prolific authors on COVID-19 vaccine publications revealed that most of these investigators had an infectious disease background with many in this category coming from influenza and HIV/AIDS research. Indeed, an effective and durable HIV vaccine and a universal influenza vaccine have been very high priority areas for many years (Fauci, 2017; Erbelding et al., 2018) given the public health impact of these diseases. It's likely that the knowledge, skills, and resources gained from decades of research in these areas helped investigators respond rapidly to a new pandemic. Interestingly, the most prolific authors with expertise in coronavirus research were not as frequent as other infectious diseases which may reflect the smaller pre-existing research community relative to other fields before the pandemic. Maybe most surprising was the frequency of top authors with expertise in cancer research. However, since the discovery of viruses that cause cancer, such as the human papilloma virus and the Epstein-Barr virus, there has been significant overlap between infectious disease research and cancer research. In addition, the development of new vaccines has long been a focus of cancer research, whether it is a vaccine targeting viruses that increase the risk of cancer or therapeutic vaccines that target cancer specific antigens (Basu et al., 2021; Saxena et al., 2021).

Another way to quantify the flow of scientific knowledge from one field to another, besides researcher expertise, is to examine what type of publications were being cited by COVID-19 vaccine articles. Here we show that coronavirus related research was cited more than any other field by far. Thus emphasizing the importance of domain specific knowledge and highlighting the need for robust research funding focused on pathogens with pandemic potential (Graham and Corbett, 2020). In addition, there was significant overlap between the most cited fields and author expertise including influenza, HIV/AIDS, and flavivirus but notable differences between the two lists include diabetes, hypertension, and tuberculosis. While the relative contributions of these research areas were less than many other categories, the connection between this work and COVID-19 vaccine research was less apparent until we used topic clustering to identify major areas of study within this broad corpus of vaccine-related articles.

Indeed, using topic clustering and tracing citations from each cluster back to specific research areas helped us determine that research related to diabetes, hypertension, and inflammation was cited frequently by articles related to the study of various risk factors for COVID-19 infection and vaccination. Additional support for the validity of this analysis came from the strong connection between a cluster of papers focused on the BCG vaccine, used for tuberculosis prevention, and prior tuberculosis research. The diversity of scientific areas that contributed to the COVID-19 vaccine response, revealed by our citation analysis, including many outside infectious disease research highlights the importance of funding and sustaining a diverse research enterprise to facilitate a rapid response to future pandemics.

It may be generally accepted that the research community's rapid response to the COVID-19 pandemic was enabled by years of previous research. With our methods, we were able to be more specific by quantifying the relative contributions of different areas of researcher expertise, the focus of prior research, and the relationships of these to different types of COVID-19 vaccine research. While some of these relationships might have been expected, others may be less apparent. The high degree of correspondence between clusters of COVID-19 publications, disease relevance of the cited research, and areas of author expertise combine to provide a coherent picture of the relationships existing in the research literature. Studies incorporating systemic and quantifiable analyses of scientific literature, like the one presented here, coupled with a thorough review of the individual researchers and key scientific breakthroughs will be essential to help understand the research foundations of our pandemic response and may help inform future pandemic preparedness efforts.

## Data availability statement

The datasets presented in this study can be found online *via* the following link: https://doi.org/10.6084/m9.figshare.21365133. The R and Python codes used to analyze the data are available upon request to the corresponding author.

## Author contributions

JN and DG conceived the study design. JN curated the COVID-19 vaccine publication data and performed the citation analysis. PS curated the authors' publication histories data and performed the subsequent analysis. JN, PS, and KM conceived the analytical methodologies. KM performed the literature clustering analysis. All authors participated in writing and editing the manuscript.
